# The Far-IR Fe–Cp Vibrations of Deuterated Ferrocene: A DFT Benchmark and Physics-Based AI Assessment

**DOI:** 10.3390/molecules31101692

**Published:** 2026-05-17

**Authors:** Feng Wang, Vladislav Vasilyev

**Affiliations:** 1 School of Science, Computing and Engineering Technologies, Swinburne University of Technology, Melbourne, VIC 3122, Australia; 2 National Computational Infrastructure, Australian National University, Canberra, ACT 0200, Australia; vladislav.vassiliev@anu.edu.au

**Keywords:** ferrocene deuteration, far-infrared spectroscopy, Fe–Cp vibrational modes, density functional theory (DFT), physics-based artificial intelligence (AI)

## Abstract

Deuteration provides a controlled perturbation for probing isotope and symmetry effects in organometallic vibrational spectra. Here, density functional theory (DFT) is used to systematically examine the evolution of far-infrared (400–600 cm^−1^) Fe–Cp vibrational modes in fully protonated, partially deuterated, and fully deuterated ferrocene. All three characteristic modes—the a_2_″ torsional mode and the two e_1_′ bending modes—exhibit monotonic red-shifts with increasing deuteration. The a_2_″ mode shows the largest isotope sensitivity, shifting by ~28 cm^−1^ across the DFT series, whereas the e_1_′ modes shift by ~11–12 cm^−1^ and undergo symmetry-dependent splitting of up to ~2 cm^−1^ under partial deuteration. These results establish the a_2_″ band as a sensitive probe of the degree of deuteration and the e_1_′ splitting as a diagnostic of symmetry reduction. A physics-based AI surrogate model reproduces the overall red-shift trends but deviates at high deuteration, with maximum errors of ~16.6 cm^−1^, highlighting the limits of reduced-mass scaling.

## 1. Introduction

Since its discovery in 1951, ferrocene (Fe(C_5_H_5_)_2_, Fc) [1,2,3] has served as a cornerstone of modern organometallic chemistry, shaping the concepts of metal–ligand bonding, molecular symmetry, and electronic structure [4,5,6,7]. While the gross structural features of Fc were established early, more subtle aspects of its vibrational behaviour—particularly those linked to conformational preference [8,9] and low-frequency dynamics [10]—continue to attract attention [7]. In the context of the 75th anniversary of ferrocene, revisiting these foundational vibrational questions with modern quantum mechanical and data-driven approaches offers an opportunity to extract new physical insight from a system often regarded as fully understood.

Among the vibrational features of ferrocene, the far-infrared (far-IR) region spanning approximately 400–500 cm^−1^ is especially informative, as it hosts collective Fe–Cp interactions and Cp ring relaxation. A characteristic band near 450–460 cm^−1^ has long been associated with the staggered Fc conformer [8], yet its detailed origin remains subtle [11]. High-resolution experiments reveal only a single band for staggered Fc [8], as the expected splitting of closely related modes lies below typical instrumental resolution. As a result, this feature represents a sensitive—yet historically under-exploited—probe of Fc symmetry and low-frequency dynamics [8].

Isotopic substitution provides a powerful means of perturbing vibrational motion without significantly altering the underlying electronic structure. In ferrocene, the replacement of hydrogen by deuterium modifies the reduced mass of Cp-based motions and, crucially, lowers the effective vibrational symmetry even when the equilibrium geometry remains staggered. Despite this diagnostic potential, the influence of deuteration on the far-IR signature of staggered Fc has received comparatively little attention, particularly in relation to symmetry-driven mode coupling and IR activity [12]. Previous studies have focused largely on fully deuterated ferrocene, Fe(C_5_D_5_)_2_ [13,14,15], leaving the partially deuterated isotopologues largely unexplored.

For staggered ferrocene, the low-frequency IR band in the 405–500 cm^−1^ region appears as a single unresolved feature under typical experimental conditions, in contrast to the characteristic splitting observed for the eclipsed conformer [8]. Upon the stepwise deuteration of the Cp hydrogens, this band is expected to shift in a systematic but non-trivial manner, reflecting reduced-mass effects while preserving the staggered framework. Because these isotope-induced shifts are incremental and internally correlated across a well-defined series of isotopologues, partially deuterated ferrocene provides a stringent benchmark for distinguishing predictive accuracy from qualitative trend capture. In this work, density functional theory (DFT) calculations at the B3LYP/m6-31G(d) level [8] establish a consistent quantum mechanical reference for the far-IR band positions of partially deuterated staggered ferrocene species, against which AI-based predictions are directly assessed.

**Figure 1:**
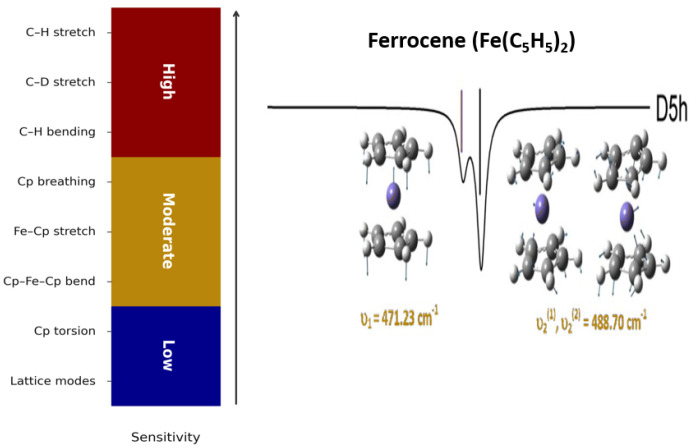
The AI—predicted isotope sensitivity of Fc vibrational modes upon partial deuteration (Fc–d_n_), which fits well to the IR spectrum of Lippincott et al. [11] and identifies the 400–600 cm^−1^ region [8,13] as an intermediate sensitivity window for isotope—and symmetry—dependent effects. The signature IR vibrations of original ferrocene are also given [8].

More recently, machine learning and data-driven approaches have been increasingly applied to structure–property relationships in ferrocene derivatives, particularly where systematic variation across chemically related systems is of interest [16,17]. Automated workflows combining high-throughput DFT and ML analysis have proven effective in identifying correlated trends and sensitivities [18], including Δ-machine learning strategies that refine DFT-level response properties such as isotropic polarisabilities and polarisability tensors [16]. Within this framework, Figure 1 provides a global isotope sensitivity map, highlighting the 400–600 cm^−1^ region as an intermediate regime where isotope effects are neither trivial nor overwhelmed by local C–H/C–D vibrations.

In this work, we focus on the diagnostically important far-IR Fe–Cp band of staggered Fc near 450–500 cm^−1^ and its response to stepwise deuteration. The B3LYP/m6-31G(d) approach is retained for consistency with prior benchmark studies of ferrocene vibrational spectroscopy, where it has been demonstrated to reproduce experimental frequencies with reasonable accuracy [8] without scaling and to provide robust trends across isotopic substitution. More recent functionals were not adopted here, as the present work focuses on relative isotope effects rather than absolute frequency refinement. The B3LYP/m6-31G(d) method produces (eclipsed, D_5h_) Fc at 471.23 cm^−1^ (a_2_″) and 488.70 cm^−1^ (e_1_′), which correspond to the measured IR bands at 472 cm^−1^ and 479 cm^−1^ (Fc crystal) and 480 cm^−1^ and 496 cm^−1^ (Fc vapour) [11]. Using DFT calculations at the B3LYP/m6-31G(d) level [8] together with physics-based AI spectral estimation, we establish a consistent theoretical reference for partially deuterated staggered ferrocene and quantify how isotopic substitution modifies this band through reduced-mass effects and symmetry breaking. The resulting DFT–AI comparison provides a physically transparent and quantitatively demanding assessment of predictive accuracy for subtle far-IR frequency shifts within a narrowly constrained vibrational manifold.

## 2. Computational and Physics-Based AI Methodology

Each of the 10 hydrogen atoms in Fc can be deuterated. Partial deuteration was modelled via stepwise H → D substitution on both the Cp rings. For each degree of deuteration, all symmetry-distinct substitution patterns were considered. This yields a total of 26 distinct staggered Fc-d_n_ isotopologues for n = 0–10, which shown in Table 1.

Geometry optimisations and harmonic vibrational frequency calculations were carried out using density functional theory (DFT) at the B3LYP [19]/m6-31G(d) level [8], following the computational protocol previously established for ferrocene conformers. The modified m6-31G(d) basis set [20] was adopted for Fe to ensure a balanced description of the 3d and 4s atomic states [20], enabling the reproduction of experimental IR frequencies in the 400–500 cm^−1^ region without empirical scaling [8]. In a previous study [21], we evaluated the conformer energy gap (ΔE = E(D_5d_) − E(D_5h_)) between the staggered (D_5d_) and eclipsed (D_5h_) forms of Fc, which serves as a sensitive benchmark for assessing the accuracy of electronic structure methods. The value of 0.58 kcal⋅mol^−1^ obtained from B3LYP/m6-31G(d)) calculations [8] is consistent with experimental estimates for gas-phase Fc [22]. In the same study, the corresponding values obtained using CAM-B3LYP/m6-31G(d), B3LYP-D3/m6-31G(d), and CAM-B3LYP-D3/m6-31G(d) were 0.60, 0.10, and 0.58 kcal·mol^−1^, respectively [21]. These results indicate that dispersion effects are property-dependent. For isolated molecules such as gas-phase Fc, long-range dispersion interactions do not provide a dominant contribution to conformer energy differences, intramolecular vibrational frequencies, or electronic properties such as UV–Vis spectra [23].

All calculations were performed in the gas phase. The un-deuterated eclipsed (D_5h_) Fc was taken as the reference structure (as staggered Fc conformer (D_5d_) is a transition state in isolation [8]). This conformer of Fc is characterised by two nearly degenerate IR-active modes splitting at 471 and 488 cm^−1^ (1:2). For each isotopologue, Fc-d_n_, vibrational frequencies and IR intensities were extracted for all modes with non-zero intensity in the 400–600 cm^−1^ region, with particular emphasis on those evolving from the parent 471/488 cm^−1^ Fe–Cp modes. All quantum mechanical calculations were performed using Gaussian 16 Computational Chemistry Package [24].

The AI predictions for the IR bands of deuterated Fc isotopologues are grounded in the harmonic oscillator isotope shift [25] rather than statistical regression against DFT data, as the aim of this study is to compare the effectiveness and accuracy of physics-based AI evaluation. For any vibrational mode, isotopic substitution leaves the electronic structure and force constant *k* unchanged while increasing the effective reduced mass μ, shifting the frequency according to the following:

(1) $$\begin{array}{cccc} & \frac{{\tilde{\nu }}_{D}}{{\tilde{\nu }}_{H}}=\sqrt{\frac{\mu_{H}}{\mu_{D}}} & & \end{array}$$

Because the IR fingerprint modes of D_5h_ Fc are collective Fe–Cp vibrations—the a_2_″ mode involving axial Fe displacement against synchronised Cp ring flips and the e_1_′ mode involving lateral Fe wobble perpendicular to the C_5_ axis [8]—the H/D atoms participate only partially in each mode. The effective mass μ therefore cannot be equated with the atomic mass of a single oscillator. Instead, the rotational constants (A, B, C in GHz) tabulated for each isotopologue in Table S1 serve as a proxy for the mass distribution change, since the moment of inertia (I) scales as I ∝ 1/B. For the a_2_″ mode, which involves axial mass redistribution, the A constant is the relevant quantity; for the e_1_′ mode, which involves in-plane motion, the perpendicular constants B and C are used. Applying the Teller–Redlich product rule approximation [25,26], the predicted band positions for each nD isotopologue are as follows:

(2) $${\tilde{\nu }}_{nD}(a_{2}^{\prime \prime })={\tilde{\nu }}_{0}(a_{2}^{\prime \prime })\times \sqrt{\frac{A_{0}}{A_{nD}}}$$

(3) $${\tilde{\nu }}_{nD}(e_{1}^{\prime })={\tilde{\nu }}_{0}(e_{1}^{\prime })\times \sqrt{\frac{B_{0}}{B_{nD}}}$$

where A_0_ = 2.19453 GHz and B_0_ = C_0_ = 1.05862 GHz are the rotational constants of the fully protonated D5h-Fc-0, and A_n_D and B_n_D are those of the nD isotopologue from Table S1. Where multiple isotopologues exist at the same nD level, the mean rotational constant across all substitution patterns is used, reflecting the statistical distribution of deuteration sites. At full deuteration (nD = 10), Equations (2) and (3) yield 424.9 cm^−1^ for the a_2_″ mode and 463.1 cm^−1^ for e_1_′, compared to the parent values of 471.23 and 488.70 cm^−1^ respectively.

For isotopologues in which asymmetric deuteration breaks the C_5_ symmetry of D_5h_ Fc, the doubly degenerate e_1_′ mode lifts into two distinct components. This is signalled by B ≠ C in the rotational constants, and the two sub-band positions are predicted as follows:

(4) $${\tilde{\nu }}_{x}={\tilde{\nu }}_{0}(e_{1}^{\prime })\times \sqrt{\frac{B_{0}}{B_{nD}}},{\tilde{\nu }}_{y}={\tilde{\nu }}_{0}(e_{1}^{\prime })\times \sqrt{\frac{B_{0}}{C_{nD}}}$$

The splitting Δ$\tilde{\nu }$ = ${\tilde{\nu }}_{y}-{\tilde{\nu }}_{x}$ vanishes at nD = 0, 5, and 10, where the molecule retains full or partial C_5_ symmetry (B = C) and reaches a maximum at substitution patterns that most strongly break the in-plane equivalence of the two wobble directions. The complete set of predicted band positions for all 26 isotopologues is given in Table S2. $\tilde{\nu }$(a_2_″) = 471.23 cm^−1^ and $\tilde{\nu }$(e_1_′) = 488.70 cm^−1^ are used as the nD = 0 reference [8], and Equation (4) is applied so that the a_2_″ shift is scaled by √(A_0_/A_n__D_), the e_1_′ shift is scaled by √(B_0_/B_nD_) and the e_1_′_γ_ shift is scaled by √(B_0_/C_nD_). The effective mass ratio for nD-Fc deuterium substitutions out of 10 H atoms can be estimated using the Teller-Redlich product rule approximation [26,27], which is given in Supplementary Materials.

The so-called “physics-based AI” model used here is a physics-derived surrogate without machine learning training. It is largely governed by reduced-mass scaling derived from rotational descriptors and consequently reproduces the main isotope effects but does not explicitly include force constant changes or vibrational coupling. Artificial Intelligence (AI) tools such as Microsoft 365 Copilot (GPT-5 chat model), ChatGPT (OpenAI’s GPT-5.5 model) and Claude (Claude Sonnet 4.6) are employed in the AI assessment.

## 3. Results and Discussion

Before analysing the far-IR signature bands in detail, it is important to clarify why vibrational modes in the 400–600 cm^−1^ region, associated with Fe–Cp and Cp–Fe–Cp collective motions, are of particular relevance for deuterated Fc. Figure 1 provides a global overview of the isotope sensitivity of ferrocene vibrational modes upon partial deuteration, as predicted by the physics-based AI model. At high frequencies, C–H stretching and bending modes exhibit large isotope-induced shifts. However, these shifts are dominated by local reduced-mass effects and therefore provide limited structural or symmetry-specific information. In contrast, low-frequency modes involving collective metal–ligand motion show more moderate isotope sensitivity but one that is strongly influenced by vibrational coupling and symmetry breaking. This intermediate sensitivity regime is particularly valuable for disentangling global mass effects from changes in molecular symmetry.

Notably, the Cp–Fe–Cp bending mode near ~470 cm^−1^ [8] falls squarely within this regime. This band was previously identified as a prominent and well-isolated feature in the classical vapour-phase IR spectrum of ferrocene reported by Lippincott and co-workers [11], despite the limited experimental resolution available at the time. Its persistence as a strong experimental signal indicates both favourable IR activity and intrinsic robustness with respect to thermal broadening and conformational averaging. As a result, this mode provides a rare opportunity to track isotope-induced frequency shifts and symmetry effects in a metal–organic framework using far-IR spectroscopy. The heatmap in Figure 1 highlights this behaviour quantitatively. While Cp–Fe–Cp and related Fe–Cp torsional motions do not exhibit the largest absolute isotope shifts, they show enhanced sensitivity to deuteration patterns and symmetry reduction compared with higher-frequency modes. This makes the 400–600 cm^−1^ region the most informative spectral window for analysing partially deuterated ferrocene isotopologues.

**Figure 2:**
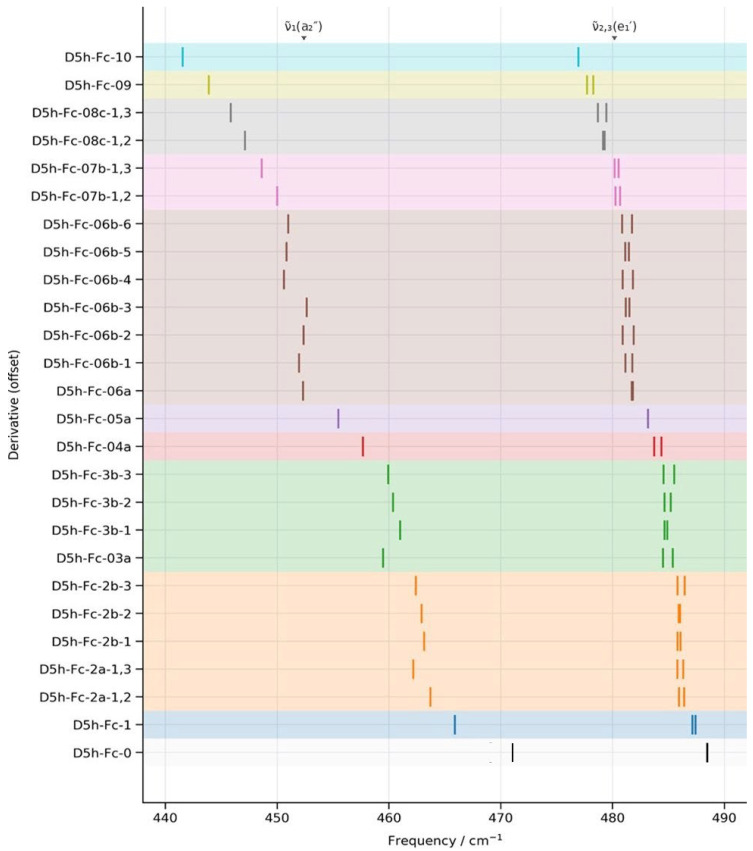
Far-IR vibrational bands (400–600 cm^−1^) for eclipsed Fc and partially deuterated Fc derivatives (nD), calculated using the DFT-based B3LYP/m6-31G(d) mode. The first band of the a_2_″ Fe–Cp torsional mode (${\tilde{\nu }}_{1}$ < ~470 cm^−1^) and the e_1_′ Fe–Cp bending modes (${\tilde{\nu }}_{2}$, ${\tilde{\nu }}_{3}$ < ~490 cm^−1^) are shown. The fully deuterated reference, D5h-Fc-0, is plotted in black (at the bottom of the spectrum).

The offset stick spectrum in Figure 2 summarises the evolution of the three characteristic far-IR bands of ferrocene—$\tilde{\nu }$_1_(a_2_″) and $\tilde{\nu }$_2,3_(e_1_′)—as a function of increasing and increasingly asymmetric deuteration. Several clear and physically meaningful trends emerge directly from the DFT calculations. Across the entire series from D_5h_-Fc-0 to D_5h_-Fc-10, all three far-IR bands exhibit a monotonic red-shift with increasing total deuteration. The a_2_″ mode $\tilde{\nu }$_1_ (Fe–Cp torsion/relaxation) shows the largest absolute shift, decreasing from ~471 cm^−1^ in D_5h_-Fc-0 [8] to ~442 cm^−1^ in D_5h_-Fc-10. The e_1_′ modes $\tilde{\nu }$_2,3_ (Fe–Cp bending) shift more moderately, from ~488 [8] cm^−1^ to ~477 cm^−1^. This behaviour reflects the fact that H→D substitution increases the effective reduced mass of motions involving the cyclopentadienyl rings while leaving metal–ligand force constants essentially unchanged. The larger isotope sensitivity of $\tilde{\nu }$_1_ is consistent with its stronger coupling to collective Cp ring motion, whereas $\tilde{\nu }$_2,3_ involve a greater Fe–Cp bending character and therefore show a smaller mass dependence.

Partial deuteration introduces an additional effect that is absent in the fully symmetric limits: the progressive lifting of the near-degeneracy of the e_1_′ modes. The DFT calculations show that even modest symmetry breaking is sufficient to split the e_1_′ pair, with the extent of splitting increasing both with total deuteration and with the asymmetry of isotope placement. This behaviour highlights the sensitivity of far-IR Fe–Cp bending modes to subtle changes in ring equivalence and confirms that the observed splittings are symmetry-driven rather than arising from changes in force constants. Importantly, the smooth evolution of the splitting across the series indicates that no mode crossing or reassignment occurs; instead, symmetry breaking continuously perturbs otherwise degenerate vibrational states.

Within groups of identical total deuteration, smaller but reproducible differences between positional isomers further illuminate the interplay between global and local isotope effects. While the overall red-shift is governed by the total number of deuterium atoms, the residual variation within each *nD* group demonstrates that the spatial distribution of isotopes modulates vibrational coupling between Cp motion and the Fe centre. This effect is the most evident for $\tilde{\nu }$_1_(a_2_″), again underscoring its collective nature, whereas the $\tilde{\nu }$_2,3_(e_1_′) modes remain comparatively insensitive to positional differences.

As a result, the DFT trends establish the 400–600 cm^−1^ region as a highly diagnostic spectral window for probing isotope effects and symmetry breaking in eclipsed Fc. Deuteration systematically tunes Fe–Cp vibrational frequencies without altering mode identity, providing a rigorous benchmark against which experimental far-IR spectra and physics-based AI predictions can be quantitatively assessed. The first band $\tilde{\nu }$_1_(a_2_″) is particularly diagnostic of total and positional deuteration, as it red-shifts more significantly than the $\tilde{\nu }$_2,3_(e_1_′) bands, whereas the latter ($\tilde{\nu }$_2,3_(e_1_′)) offers a direct probe of symmetry breaking induced by selective isotope substitution. The smooth and interpretable evolution observed here further supports the use of isotopic substitution as a precise tool for disentangling torsional and bending contributions in organometallic vibrational spectroscopy. These trends provide a rigorous quantum mechanical foundation for interpreting the experimental far-IR spectra of isotopically labelled Fc and form the benchmark against which AI-based predictions can later be assessed.

**Figure 3:**
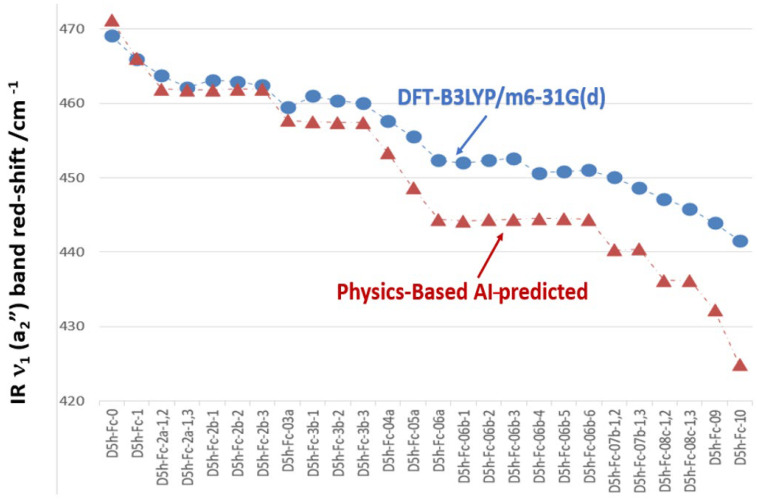
A comparison of the red—shifted IR signature of the first band ${\tilde{\nu }}_{1}$(a_2_″) of eclipsed Fc due to deuteration calculated using DFT (B3LYP/m6-31G(d)) and physically based AI prediction.

**Figure 4:**
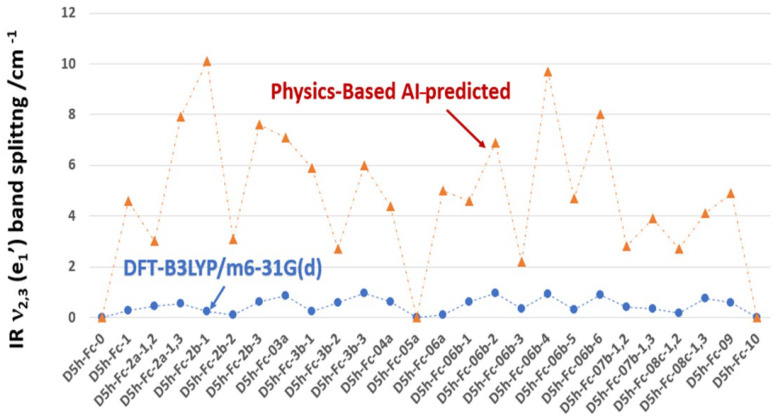
DFT—calculated and AI-predicted D_5h_ Fc e_1_′ band splitting due to deuteration. Note that at nD = 0, 5 and 10, the bands of D_5h_ Fc do not split.

The three DFT-calculated and AI-predicted IR signature vibrational bands in the 400–600 cm^−1^ region of deuterated Fc derivatives are shown in Table 2. For un-deuterated eclipsed Fc (n = 0), the DFT-based B3LYP/m6-31G(d) model predicts two closely spaced IR-active modes at ~471 and ~488 cm^−1^ [8]. The calculated splitting is approximately 17 cm^−1^ and was confirmed in high-resolution FTIR spectra [10]. This behaviour is fully consistent with earlier experimental [11] and theoretical work [8] and serves as the anchor for analysing isotope-induced perturbations. For clarity, Table 2 reports both DFT and AI values; key trends are summarised graphically in Figure 2, Figure 3 and Figure 4.

Within each nD level, isotopologues sharing the same number of deuterium substitutions but differing in their substitution pattern show measurably different band positions, demonstrating that the positional distribution of deuterium across the two Cp rings matters as much as the total count. This arises because the overlap between the D-atom positions and the displacement vectors of the a_2_″ normal mode varies depending on where on the rings the substitution occurs—D atoms placed at positions of high vibrational amplitude perturb μ_eff_ more strongly than those at near-nodal positions. This is the most clearly visible at nD = 6, where seven distinct isotopologues are represented, as shown in Table 2; the DFT-calculated $\tilde{\nu }$_1_(a_2_″) spans from 450.61 cm^−1^ (D5h-Fc-06b-4) to 452.63 cm^−1^ (D5h-Fc-06b-3), a within-nD spread of 2.02 cm^−1^, and similarly at ND = 3, the four isotopologues range from 459.46 cm^−1^ (D5h-Fc-03a) to 460.99 cm^−1^ (D5h-Fc-3b-1), a spread of 1.53 cm^−1^. This positional sensitivity means that ND alone cannot uniquely determine the band position and that isotopologues with D atoms concentrated on one ring (such as D_5h_-Fc-05a at nD = 5, where one ring is fully deuterated) behave differently from those with the same nD distributed symmetrically, linking the spectral position directly to molecular topology rather than bulk composition.

Table 2 reports that the a_2_″ band (ν_1_) red-shifts approximately twice as steeply per deuterium substitution as the e_1_′ bands (see), a differential sensitivity that has direct consequences for the persistence and shape of the D_5h_ fingerprint across the isotopologue series. Because the a_2_″ and e_1_′ modes involve different vibrational characters—axial ring-flip versus lateral Fe wobble—their H/D displacement amplitudes differ, so their sensitivity to deuteration is unequal. Concretely, across the DFT series, the a_2_″ band shifts by 27.5 cm^−1^ in total, while the e_1_′ centroid shifts by only approximately 11.5 cm^−1^, meaning that the gap between the two band clusters grows from ~19.4 cm^−1^ at nD = 0 to ~35.4 cm^−1^ at nD = 10. This widening gap is experimentally significant: rather than the two-band fingerprint collapsing or becoming ambiguous under deuteration, it in fact becomes more spread out and potentially easier to resolve at high nD, provided the instrument resolution is sufficient. This links back to the utility of the fingerprint as a conformer diagnostic—the eclipsed D_5h_ character is not erased by deuteration but is preserved and even accentuated in the separation between its two signature bands.

The $\tilde{\nu }$_1_(a_2_″) mode of the eclipsed D_5h_ Fc fingerprint region is plotted across all isotopologues in order of increasing deuteration (nD = 0 to 10), comparing DFT-calculated (blue circles) and AI-predicted (red triangles) band positions. Figure 3 reports the red-shifted IR signature bands of eclipsed Fc due to deuteration calculated using DFT (B3LYP/m6-31G(d)) and physically based AI prediction. Both the DFT-calculated and AI-predicted $\tilde{\nu }$_1_(a_2_″) bands of D_5h_ Fc shift monotonically to a lower wavenumber as the degree of deuteration increases from nD = 0 to nD = 10. This red-shift is a direct consequence of the harmonic oscillator relationship $\tilde{\nu }$ ∝ (k/μ_eff_)^(1/2)^ in which isotopic substitution leaves the force constant k unchanged but increases the effective reduced mass μ_eff_ of the mode. For the a_2_″ mode specifically, this mass sensitivity is amplified by the nature of the vibration—axial Fe motion against synchronised Cp ring flips [8]—which assigns large displacement amplitudes to the peripheral H/D atoms, making μ_eff_ strongly dependent on their mass. For example, the DFT-calculated $\tilde{\nu }$_1_(a_2_″) descends from 471 cm^−1^ at nD = 0 [8] to 441.53 cm^−1^ at nD = 10, a total red-shift of 27.5 cm^−1^ (blue dots in Figure 3), while the AI prediction spans a larger range from 471 to 425 cm^−1^, a shift of 46 cm^−1^ over the same substitution range. This common downward trend across both methods confirms that the reduced-mass effect dominates the isotopologue dependence of this mode, establishing deuteration as a predictable and systematic perturbation of the D_5h_ fingerprint.

The two methods, DFT and AI, agree closely at low deuteration (nD ≤ 3) but diverge progressively at higher nD, with the AI predictions underestimating the band position by up to 16.6 cm^−1^ at full deuteration. The AI approach relies on rotational constant scaling to estimate effective mass changes under the harmonic oscillator approximation, which treats the potential energy surface as purely quadratic and the isotope effect as a simple reduced-mass ratio. This approximation is well-founded for small perturbations but systematically neglects several physically important contributions that grow with cumulative deuterium loading. First, anharmonicity—the deviation of the true C–H/D and Fe–Cp potential from a perfect parabola—means that the vibrational frequency depends not only on the curvature at the minimum but also on higher-order terms, which respond differently to H and D substitution because D occupies a lower, narrower region of the same anharmonic well. Second, zero-point energy (ZPE) differences between C–H and C–D oscillators are substantial: the ZPE of a C–H stretch (~1750 cm^−1^ × 1/2 hc) is nearly √2 times larger than that of C–D, so progressive deuteration shifts the vibrational ground state to a meaningfully different region of the potential, effectively altering the curvature experienced by the Fe–Cp modes that are coupled to it. Third, mode mixing between the a_2_″ mode and neighbouring vibrational modes of similar symmetry becomes increasingly significant as the cumulative mass loading reshuffles the normal mode ordering and changes the degree of mechanical coupling between the Cp ring motions and the central Fe atom displacement.

None of these contributions are captured by the rotational constant scaling method, which treats the molecule as a rigid rotor with modified masses rather than a coupled anharmonic oscillator system. For example, at nD = 0, the two methods agree to within 2.1 cm^−1^ (DFT: 469.07 cm^−1^, AI: 471.2 cm^−1^ for D5h-Fc-0), and through nD = 1–4, the AI values remain within 3–5 cm^−1^ of the DFT reference, confirming that the harmonic reduced-mass approximation is adequate when only a small fraction of the ten H atoms in Fc are substituted and the perturbation to the normal mode structure is minor. However, from nD = 6 onward, the gap widens sharply and non-linearly: at nD = 7, the discrepancy reaches 8–9 cm^−1^ (D5h-Fc-07b-1,2: DFT 449.99 cm^−1^ vs. AI 440.3 cm^−1^), and at nD = 10 in fully deuterated Fc (D5h-Fc-10), it reaches 16.6 cm^−1^ (DFT: 441.53 cm^−1^ vs. AI: 424.9 cm^−1^), precisely where anharmonic, ZPE, and mode-mixing contributions are collectively the largest. This systematic and accelerating divergence confirms that the rotational constant scaling method is not transferable across the full substitution range of Fc isotopologues and links directly to the well-known breakdown of first-order mass perturbation theory when the isotopic mass change is no longer small relative to the total effective modal mass of the vibration.

The degeneracy of the e_1_′ mode is lifted upon the asymmetric deuteration of Fc, producing a non-zero Δ(e_1_′) splitting that both the DFT and AI methods predict but whose magnitude the AI approach overestimates by up to two orders of magnitude, as reported in Figure 4. When the full C_5_ rotational symmetry of D_5h_ Fc is intact, the two components of the doubly degenerate e_1_′ mode—the Fe wobble along x and along y—are energetically equivalent and collapse to a single band [8]. Partial deuteration that breaks this C_5_ equivalence removes the degeneracy constraint, allowing the force constants experienced along the two perpendicular wobble directions to differ, producing a measurable splitting Δ(e_1_′) = $\tilde{\nu }$_3_ − $\tilde{\nu }$_2_. The DFT calculations capture this effect with physically realistic magnitudes: Δ(e_1_′) remains small throughout, ranging from 0.00 cm^−1^ at the symmetric isotopologues (D5h-Fc-0, D5h-Fc-05a, and D5h-Fc-10, where C_5_ symmetry is fully or partially restored) to a maximum of only 0.97 cm^−1^ at D5h-Fc-08c-1,3, reflecting the genuinely weak perturbation that deuterium substitution exerts on the lateral force constants of the Fe–Cp framework. This small magnitude is physically reasonable: the e_1_′ force constant is dominated by the Fe–Cp interaction, which is largely insensitive to whether the Cp periphery carries H or D.

By contrast, the AI predictions show Δ(e_1_′) values that are larger by a factor of roughly 10–40, with the splitting reaching 10.1 cm^−1^ for D5h-Fc-2b-1 and 9.7 cm^−1^ for D5h-Fc-06b-4, compared to DFT values of 0.24 cm^−1^ and 0.92 cm^−1^ respectively for the same isotopologues. This gross overestimation arises because the rotational constant scaling method equates the asymmetry in the moment of inertia tensor—captured by the B − C difference—with an asymmetry in the vibrational force constants, which is physically unjustified. The moment of inertia reflects the bulk geometric mass distribution of the entire molecule, whereas the e_1_′ force constant perturbation depends only on the local change in the restoring force along each wobble direction, which is an order of magnitude smaller. Furthermore, the AI predictions show a highly irregular, spiky pattern across the isotopologue series—for instance jumping from 10.1 cm^−1^ at D5h-Fc-2b-1 down to 3.1 cm^−1^ at D5h-Fc-2b-2 and back up to 7.6 cm^−1^ at D5h-Fc-2b-3—whereas the DFT values vary smoothly and modestly between 0.11 and 0.97 cm^−1^ across the same ND = 2 isotopologues. This erratic behaviour of the AI predictions directly reflects the sensitivity of the B − C rotational constant difference to the precise geometric placement of D atoms, a sensitivity that is real at the level of molecular geometry but does not translate proportionally into force constant asymmetry. As a result, Figure 4 demonstrates that while the AI method correctly identifies which isotopologues are degenerate—recovering Δ = 0 at nD = 0, 5, and 10 in agreement with DFT—it is fundamentally unsuitable for predicting the quantitative magnitude of e_1_′ splitting, and the DFT-calculated values must be used as the reference for any experimental assignment of partially deuterated Fc isotopologues in this spectral region.

## 4. Conclusions

This study establishes a DFT benchmark for the isotope dependence of the far-IR vibrational spectrum of eclipsed Fc in the 400–600 cm^−1^ conformation signature region. Progressive deuteration induces smooth, monotonic red-shifts in the ν_1_(a_2_″) Fe–Cp torsional mode and the ν_2,3_(e_1_′) Fe–Cp bending modes while preserving their vibrational character across all isotopologues. The ν_1_(a_2_″) mode exhibits strong isotope sensitivity, red-shifting by ~28 cm^−1^ over the full series, whereas the degenerated ν_2,3_(e_1_′) modes in nD = 0 show smaller red-shifts but clear symmetry-dependent splitting under partial deuteration. Partial and asymmetric isotope substitution systematically lifts the near-degeneracy of the ν_2,3_(e_1_′) modes present in the D_5h_ limits. The magnitude of this splitting increases with both the degree and pattern of deuteration while remaining continuous across the series, indicating symmetry-driven perturbation rather than mode mixing or reassignment. Within each deuteration class, smaller yet reproducible differences between positional isomers further demonstrate that total isotope content governs the overall frequency scale, while isotope distribution modulates local vibrational coupling.

Comparison with a physics-informed AI model shows that AI reproduces the dominant DFT trends, including monotonic red-shifting and regions of enhanced splitting. However, quantitative agreement deteriorates beyond moderate deuteration (nD > 3), with deviations reaching ~16.6 cm^−1^ at full deuteration and the systematic overestimation of ν_2,3_(e_1_′) splitting. These discrepancies arise from the intrinsic limitations of reduced-mass scaling, which neglects quantum mechanical factors such as explicit force constant perturbations, anharmonicity, zero-point energy differences, and increased mode mixing at high isotope loading. Importantly, AI errors remain bounded and chemically interpretable because the model preserves parent mode identity and scales only the observed isotope response rather than extrapolating force constants. AI therefore functions as a robust trend analysis and sensitivity tool, whereas DFT remains essential for quantitative frequency prediction and the reliable assignment of closely spaced modes.

From an experimental perspective, the large and monotonic red-shift in the ν_1_(a_2_″) band provides a semi-quantitative marker of average deuteration. The persistent frequency gap between the ν_2_(e_1_′) and ν_3_(e_1_′) bands confirms that the far-IR fingerprint of the eclipsed D_5h_ conformer is retained across the full deuteration series. This work defines the limits and complementarity of DFT and physics-based AI for interpreting isotope effects in organometallic far-IR spectroscopy. The present approach is expected to lose reliability in systems involving strong anharmonicity, significant structural perturbations, or heavy substitution, where reduced-mass scaling is no longer sufficient to describe vibrational behaviour.

## Figures and Tables

**Table 1:** Deuteration levels and DFT output labels for D_5h_ Fc isotopologues with available IR band positions.

Deuteration Level	Meaning	DFT Output Labels *	Cals [8] $\tilde{\nu }$_1_(a_2_″)/$\tilde{\nu }$_2,3_(e′)	Expt [11,13] ^#^ $\tilde{\nu }$_1_(a_2_″)/$\tilde{\nu }$_2,3_(e′)
0D	Fully protonated Fc	D5h-Fc-0	471.23/488.70	480/496 482.04/500.90
1D	Mono-deuterated Fc	D5h-Fc-1		
2D	Di-deuterated Fc	D5h-Fc-2a-1,2; D5h-Fc-2a-1,3; D5h-Fc-2b-1; D5h-Fc-2b-2; D5h-Fc-2b-3		
3D	Tri-deuterated Fc	D5h-Fc-03a; D5h-Fc-3b-1; D5h-Fc-3b-2; D5h-Fc-3b-3		
4D	Tetra-deuterated Fc	D5h-Fc-04a		
5D	Penta-deuterated Fc	D5h-Fc-05a		
6D	Hexa-deuterated Fc	D5h-Fc-06a; D5h-Fc-06b-1; D5h-Fc-06b-2; D5h-Fc-06b-3; D5h-Fc-06b-4; D5h-Fc-06b-5; D5h-Fc-06b-6		
7D	Hepta-deuterated Fc	D5h-Fc-07b-1,2; D5h-Fc-07b-1,3		
8D	Octa-deuterated Fc	D5h-Fc-08c-1,2; D5h-Fc-08c-1,3		
9D	Nona-deuterated Fc	D5h-Fc-09		453.87/479.29/482.35
10D	Fully deuterated Fc	D5h-Fc-10		449.87/485.78/488.30

* a—same Cp; b—different Cp. This ordering reflects actual isotope substitution count, not filename order. ^#^ at temperature of 388 K [13].

**Table 2:** A comparison of the DFT-calculated and AI-predicted IR signature vibrational bands in the 400–600 cm^−1^ region of deuterated Fc derivatives (cm^−1^) *.

nD	Derivative	DFT Calculated				AI Estimated			
		$\tilde{\nu }$_1_(a_2_″)	$\tilde{\nu }$_2_(e_1_′)	$\tilde{\nu }$_3_(e_1_′)	Δ(e_1_′)	$\tilde{\nu }$_1_(a_2_″)	$\tilde{\nu }$_2_(e_1_′)	$\tilde{\nu }$_3_(e_1_′)	Δ(e_1_′)
**0**	**D5h-Fc-0**	471.00	*488.00*	*488.00*	*0.00*	471.00	*488.00*	*488.00*	*0.00*
**1**	**D5h-Fc-1**	465.89	487.13	487.40	**0.27**	466.00	483.40	488.00	**4.60**
**2**	**D5h-Fc-2a-1,2**	463.68	485.93	486.38	**0.45**	461.90	481.40	484.40	**3.00**
**2**	**D5h-Fc-2a-1,3**	462.15	485.78	486.31	**0.54**	461.80	478.50	486.40	**7.90**
**2**	**D5h-Fc-2b-1**	463.14	485.79	486.04	**0.24**	461.80	477.60	487.70	**10.10**
**2**	**D5h-Fc-2b-2**	462.90	485.91	486.03	**0.11**	461.90	481.30	484.40	**3.10**
**2**	**D5h-Fc-2b-3**	462.39	485.81	486.43	**0.63**	461.90	478.80	486.40	**7.60**
**3**	**D5h-Fc-03a**	459.46	484.51	485.36	**0.85**	457.70	476.40	483.50	**7.10**
**3**	**D5h-Fc-3b-1**	460.99	484.63	484.87	**0.24**	457.50	477.10	483.00	**5.90**
**3**	**D5h-Fc-3b-2**	460.35	484.62	485.19	**0.57**	457.40	478.80	481.50	**2.70**
**3**	**D5h-Fc-3b-3**	459.92	484.55	485.50	**0.95**	457.40	477.40	483.40	**6.00**
**4**	**D5h-Fc-04a**	457.66	483.72	484.34	**0.61**	453.30	474.60	479.00	**4.40**
**5**	**D5h-Fc-05a**	455.47	*483.15*	*483.15*	*0.00*	448.60	*472.30*	*472.30*	*0.00*
**6**	**D5h-Fc-06a**	452.30	481.69	481.80	**0.11**	444.40	468.50	473.50	**5.00**
**6**	**D5h-Fc-06b-1**	451.94	481.14	481.75	**0.61**	444.20	468.80	473.40	**4.60**
**6**	**D5h-Fc-06b-2**	452.35	480.89	481.87	**0.97**	444.30	467.30	474.20	**6.90**
**6**	**D5h-Fc-06b-3**	452.63	481.15	481.49	**0.33**	444.40	469.90	472.10	**2.20**
**6**	**D5h-Fc-06b-4**	450.61	480.89	481.81	**0.92**	444.50	465.70	475.40	**9.70**
**6**	**D5h-Fc-06b-5**	450.83	481.12	481.44	**0.32**	444.50	468.70	473.40	**4.70**
**6**	**D5h-Fc-06b-6**	450.97	480.83	481.71	**0.89**	444.40	466.90	474.90	**8.00**
**7**	**D5h-Fc-07b-1,2**	449.99	480.24	480.65	**0.40**	440.30	465.90	468.70	**2.80**
**7**	**D5h-Fc-07b-1,3**	448.60	480.16	480.52	**0.36**	440.40	463.30	467.20	**3.90**
**8**	**D5h-Fc-08c-1,2**	447.12	479.12	479.29	**0.18**	436.20	462.80	465.50	**2.70**
**8**	**D5h-Fc-08c-1,3**	445.83	478.67	479.42	**0.75**	436.20	460.60	464.70	**4.10**
**9**	**D5h-Fc-09**	443.88	477.69	478.26	**0.57**	432.20	458.60	463.50	**4.90**
**10**	**D5h-Fc-10**	441.53	*476.94*	*476.94*	*0.00*	424.90	*463.10*	*463.10*	*0.00*

* AI tools are used to help formatting this table based on the provided results. Bold indicates titles and the differences. The italics mean D5h-Fc-0, D5h-Fc-05a and D5h-Fc-10.

## Data Availability

The original contributions presented in this study are included in the article/Supplementary Material. Further inquiries can be directed to the corresponding author.

## Supplementary Materials

The following supporting information can be downloaded at https://www.mdpi.com/article/10.3390/molecules31101692/s1, Table S1: Optimized geometries of deuterated Fc and their rotational constants using DFT B3LYP/m6-31G(d) (Å); Table S2: DFT-calculated far-IR modes (400–600 cm^−1^) for selected nD-Fc (cm^−1^); Table S3: DFT calculated IR three signature vibrational bands in the 400–600 cm^−1^ region of deuterated Fc derivatives (cm^−1^); Table S4: The physics based AI-predicted IR three vibrational bands in the 400–600 cm^−1^ region of deuterated Fc derivatives (cm^−1^).
