# Supplementary material for: The Far-IR Fe–Cp Vibrations of Deuterated Ferrocene: A DFT Benchmark and Physics-Based AI Assessment
Source: Molecules. 2026 May 17;31(10):1692. doi: 10.3390/molecules31101692 (PMC13209929; doi:10.3390/molecules31101692)
Supplement: Supplementary file 1 [file molecules-31-01692-s001.zip › molecules-4273763-supplementary.pdf]

The Far-IR Fe–Cp Vibrations of Deuterated Ferrocene: A DFT Benchmark and Physics-Based AI Assessment

Feng Wang[1] and Vladislav Vasilyev[2]*

[1]School of Science, Computing and Engineering Technologies, Swinburne University of Technology, Melbourne, Victoria 3122, Australia

[2]National Computational Infrastructure, Australian National University, Canberra, ACT 0200, Australia

*Corresponding author: fwang@swin.edu.au

Supplementary materials

1. The effective mass in the AI calculations

The effective mass ratio for nD-Fc deuterium substitutions out of 10 H atoms can be estimated using the Teller-Redlich product rule approximation [1, 2]. For a mode involving partial H/D participation, the frequency ratio is:

$$\frac{\tilde{\nu}_{nD}}{\tilde{\nu}_{0D}} = \sqrt{\frac{\mu_{\mathrm{eff},H}}{\mu_{\mathrm{eff},nD}}}$$

The effective mass for each mode changes as:

$$\mu_{\mathrm{eff},nD} = \mu_{\mathrm{eff},H} + n \cdot \Delta m \cdot \alpha_{\mathrm{mode}}$$

where $\Delta m = m_D - m_H = 1.00628$ u, and α_{mode} is the H-displacement weighting factor for that mode (how much each H participates). This is extracted from the rotational constant shifts. For the parent (nD-Fc=0): $B_0 = C_0 = 1.05862$ GHz. The moment of inertia $I_0 \propto 1/B_0$. For nD-Fc=10 (fully deuterated, D_{5h}-Fc-10): $B_{10} = C_{10} = 0.95057$ GHz. The total mass added = 10 × 1.00628 u = 10.063 u. The increase in the perpendicular moment of inertia:

$$\frac{\Delta I}{I_0} = \frac{B_0 - B_{10}}{B_{10}} = \frac{1.05862 - 0.95057}{0.95057} = \frac{0.10805}{0.95057} = 0.1137$$

The H atoms sit at $r_H \approx 2.297$ Å from the C_5 axis (from the xyz coordinates: the H positions have x,y coordinates giving $\sqrt{(2.297^2)} \approx 2.297$ Å in the top ring, and similarly for others, projected radially).

For the a_2'' mode at 471 cm^{-1} — axial Fe+Cp flip [3]: This mode has Fe moving along z, with both Cp rings flipping. The H atoms move significantly (they are on the periphery of the flipping rings). From the normal mode description and the IR intensity pattern, the H-participation factor α_a_2'' is moderate. Using the A rotational constant change (which tracks axial mass distribution): $A_0 = 2.19453$ GHz (nD=0); $A_{10} = 1.7833$ GHz (nD=10),

$$\frac{\Delta A}{A_0} = \frac{2.19453 - 1.7833}{2.19453} = 0.1875$$

This larger fractional change in A vs. B confirms the H atoms contribute more to axial-perpendicular motion — consistent with the ring-flip character of a_2''.

Effective mass ratio (0d-Fc→10 d-Fc):

$$r_A = \frac{A_0}{A_{10}} = \frac{2.19453}{1.7833} = 1.2306$$

Since $I_A \propto 1/A$, and for the vibrational effective mass we use:

$$\frac{\mu_{10D}}{\mu_{0D}} |_{a_2''} \approx \frac{I_{A,10D}}{I_{A,0D}} = \frac{A_0}{A_{10}} = 1.2306$$

Predicted frequency ratio for full deuteration:

$$\frac{\tilde{\nu}_{10D}}{\tilde{\nu}_{0D}} = \frac{1}{\sqrt{1.2306}} = \frac{1}{1.1093} = 0.9015$$

Fully deuterated a_2'' = 471.23 × 0.9015 = 424.9 cm^{-1}.

For the e_1' mode (488.70 cm^{-1}) — Fe wobble (perpendicular) [3]: This mode involves Fe moving in the xy-plane, so the perpendicular (B/C) rotational constants are more relevant:

$$\frac{B_0}{B_{10}} = \frac{1.05862}{0.95057} = 1.1137$$

$$\frac{\tilde{\nu}_{10D}}{\tilde{\nu}_{0D}} = \frac{1}{\sqrt{1.1137}} = \frac{1}{1.0553} = 0.9475$$

Fully deuterated e_1' = 488.70 × 0.9475 = 463.1 cm^{-1}.

Table S1. Optimized geometries of deuterated Fc and their rotational constants using DFT B3LYP/m6-31G(d) (Å).

**Deuterated Fc**	**nD**	**Optimised geometry (xyz)**
D5h-Fc-0	0	1 26 0 0.000000 0.000000 0.000000<br>2 6 0 0.000000 1.214857 1.669565<br>3 6 0 -0.714075 -0.982840 1.669565<br>4 6 0 0.714075 -0.982840 1.669565<br>5 6 0 1.155398 0.375411 1.669565<br>6 6 0 -1.155398 0.375411 1.669565<br>7 6 0 0.000000 1.214857 -1.669565<br>8 6 0 -0.714075 -0.982840 -1.669565<br>9 6 0 0.714075 -0.982840 -1.669565<br>10 6 0 1.155398 0.375411 -1.669565<br>11 6 0 -1.155398 0.375411 -1.669565<br>12 1 0 0.000000 2.297208 1.659531<br>13 1 0 -1.350265 -1.858480 1.659531<br>14 1 0 1.350265 -1.858480 1.659531<br>15 1 0 2.184775 0.709876 1.659531<br>16 1 0 -2.184775 0.709876 1.659531<br>17 1 0 0.000000 2.297208 -1.659531<br>18 1 0 -1.350265 -1.858480 -1.659531<br>19 1 0 1.350265 -1.858480 -1.659531<br>20 1 0 2.184775 0.709876 -1.659531<br>21 1 0 -2.184775 0.709876 -1.659531<br><br>Rotational constants (GHZ):<br>2.1945345 1.0586192 1.0586192
D5h-Fc-1	1	1 26 0 0.000000 0.000000 0.000000<br>2 6 0 0.000000 1.214857 1.669565<br>3 6 0 -0.714075 -0.982840 1.669565<br>4 6 0 0.714075 -0.982840 1.669565<br>5 6 0 1.155398 0.375411 1.669565<br>6 6 0 -1.155398 0.375411 1.669565<br>7 6 0 0.000000 1.214857 -1.669565<br>8 6 0 -0.714075 -0.982840 -1.669565<br>9 6 0 0.714075 -0.982840 -1.669565<br>10 6 0 1.155398 0.375411 -1.669565<br>11 6 0 -1.155398 0.375411 -1.669565<br>12 1 0 0.000000 2.297208 1.659531<br>13 1 0 -1.350265 -1.858480 1.659531<br>14 1 0 1.350265 -1.858480 1.659531<br>15 1 0 2.184775 0.709876 1.659531<br>16 1 0 -2.184775 0.709876 1.659531<br>17 1 0 0.000000 2.297208 -1.659531<br>18 1 0 -1.350265 -1.858480 -1.659531<br>19 1 0 1.350265 -1.858480 -1.659531<br>20 1 0 2.184775 0.709876 -1.659531<br>21 1 0 -2.184775 0.709876 -1.659531<br><br>Rotational constants (GHZ):<br>2.1458732 1.0524115 1.0410898
D5h-Fc-2a-1,2	2	1 26 0 0.000000 0.000000 0.000000<br>2 6 0 0.000000 1.214857 1.669565<br>3 6 0 -0.714075 -0.982840 1.669565

		4 6 0 0.714075 -0.982840 1.669565<br>5 6 0 1.155398 0.375411 1.669565<br>6 6 0 -1.155398 0.375411 1.669565<br>7 6 0 0.000000 1.214857 -1.669565<br>8 6 0 -0.714075 -0.982840 -1.669565<br>9 6 0 0.714075 -0.982840 -1.669565<br>10 6 0 1.155398 0.375411 -1.669565<br>11 6 0 -1.155398 0.375411 -1.669565<br>12 1 0 0.000000 2.297208 1.659531<br>13 1 0 -1.350265 -1.858480 1.659531<br>14 1 0 1.350265 -1.858480 1.659531<br>15 1 0 2.184775 0.709876 1.659531<br>16 1 0 -2.184775 0.709876 1.659531<br>17 1 0 0.000000 2.297208 -1.659531<br>18 1 0 -1.350265 -1.858480 -1.659531<br>19 1 0 1.350265 -1.858480 -1.659531<br>20 1 0 2.184775 0.709876 -1.659531<br>21 1 0 -2.184775 0.709876 -1.659531<br><br>Rotational constants (GHZ):<br>2.0997733 1.0383777 1.0319023
D5h-Fc-2a-1,3	2	1 26 0 0.000000 0.000000 0.000000<br>2 6 0 0.000000 1.214857 1.669565<br>3 6 0 -0.714075 -0.982840 1.669565<br>4 6 0 0.714075 -0.982840 1.669565<br>5 6 0 1.155398 0.375411 1.669565<br>6 6 0 -1.155398 0.375411 1.669565<br>7 6 0 0.000000 1.214857 -1.669565<br>8 6 0 -0.714075 -0.982840 -1.669565<br>9 6 0 0.714075 -0.982840 -1.669565<br>10 6 0 1.155398 0.375411 -1.669565<br>11 6 0 -1.155398 0.375411 -1.669565<br>12 1 0 0.000000 2.297208 1.659531<br>13 1 0 -1.350265 -1.858480 1.659531<br>14 1 0 1.350265 -1.858480 1.659531<br>15 1 0 2.184775 0.709876 1.659531<br>16 1 0 -2.184775 0.709876 1.659531<br>17 1 0 0.000000 2.297208 -1.659531<br>18 1 0 -1.350265 -1.858480 -1.659531<br>19 1 0 1.350265 -1.858480 -1.659531<br>20 1 0 2.184775 0.709876 -1.659531<br>21 1 0 -2.184775 0.709876 -1.659531<br><br>Rotational constants (GHZ):<br>2.0980730 1.0444279 1.0261375
D5h-Fc-2b-1	2	1 26 0 0.000000 0.000000 0.000000<br>2 6 0 0.000000 1.214857 1.669565<br>3 6 0 -0.714075 -0.982840 1.669565<br>4 6 0 0.714075 -0.982840 1.669565<br>5 6 0 1.155398 0.375411 1.669565<br>6 6 0 -1.155398 0.375411 1.669565<br>7 6 0 0.000000 1.214857 -1.669565<br>8 6 0 -0.714075 -0.982840 -1.669565<br>9 6 0 0.714075 -0.982840 -1.669565<br>10 6 0 1.155398 0.375411 -1.669565<br>11 6 0 -1.155398 0.375411 -1.669565

		12 1 0 0.000000 2.297208 1.659531<br>13 1 0 -1.350265 -1.858480 1.659531<br>14 1 0 1.350265 -1.858480 1.659531<br>15 1 0 2.184775 0.709876 1.659531<br>16 1 0 -2.184775 0.709876 1.659531<br>17 1 0 0.000000 2.297208 -1.659531<br>18 1 0 -1.350265 -1.858480 -1.659531<br>19 1 0 1.350265 -1.858480 -1.659531<br>20 1 0 2.184775 0.709876 -1.659531<br>21 1 0 -2.184775 0.709876 -1.659531<br><br>Rotational constants (GHZ):<br>2.0987784 1.0464694 1.0241870
D5h-Fc-2b-2	2	1 26 0 0.000000 0.000000 0.000000<br>2 6 0 0.000000 1.214857 1.669565<br>3 6 0 -0.714075 -0.982840 1.669565<br>4 6 0 0.714075 -0.982840 1.669565<br>5 6 0 1.155398 0.375411 1.669565<br>6 6 0 -1.155398 0.375411 1.669565<br>7 6 0 0.000000 1.214857 -1.669565<br>8 6 0 -0.714075 -0.982840 -1.669565<br>9 6 0 0.714075 -0.982840 -1.669565<br>10 6 0 1.155398 0.375411 -1.669565<br>11 6 0 -1.155398 0.375411 -1.669565<br>12 1 0 0.000000 2.297208 1.659531<br>13 1 0 -1.350265 -1.858480 1.659531<br>14 1 0 1.350265 -1.858480 1.659531<br>15 1 0 2.184775 0.709876 1.659531<br>16 1 0 -2.184775 0.709876 1.659531<br>17 1 0 0.000000 2.297208 -1.659531<br>18 1 0 -1.350265 -1.858480 -1.659531<br>19 1 0 1.350265 -1.858480 -1.659531<br>20 1 0 2.184775 0.709876 -1.659531<br>21 1 0 -2.184775 0.709876 -1.659531<br><br>Rotational constants (GHZ):<br>2.0991479 1.0385784 1.0316053
D5h-Fc-2b-3	2	1 26 0 0.000000 0.000000 0.000000<br>2 6 0 0.000000 1.214857 1.669565<br>3 6 0 -0.714075 -0.982840 1.669565<br>4 6 0 0.714075 -0.982840 1.669565<br>5 6 0 1.155398 0.375411 1.669565<br>6 6 0 -1.155398 0.375411 1.669565<br>7 6 0 0.000000 1.214857 -1.669565<br>8 6 0 -0.714075 -0.982840 -1.669565<br>9 6 0 0.714075 -0.982840 -1.669565<br>10 6 0 1.155398 0.375411 -1.669565<br>11 6 0 -1.155398 0.375411 -1.669565<br>12 1 0 0.000000 2.297208 1.659531<br>13 1 0 -1.350265 -1.858480 1.659531<br>14 1 0 1.350265 -1.858480 1.659531<br>15 1 0 2.184775 0.709876 1.659531<br>16 1 0 -2.184775 0.709876 1.659531<br>17 1 0 0.000000 2.297208 -1.659531<br>18 1 0 -1.350265 -1.858480 -1.659531<br>19 1 0 1.350265 -1.858480 -1.659531

		20 1 0 2.184775 0.709876 -1.659531<br>21 1 0 -2.184775 0.709876 -1.659531<br><br>Rotational constants (GHZ):<br>2.0997905 1.0438277 1.0260594
D5h-Fc-b-03a	3	1 26 0 -0.000000 -0.000000 -0.000000<br>2 6 0 -1.214857 -0.000000 -1.669565<br>3 6 0 0.982840 0.714075 -1.669565<br>4 6 0 0.982840 -0.714075 -1.669565<br>5 6 0 -0.375412 -1.155398 -1.669565<br>6 6 0 -0.375412 1.155398 -1.669565<br>7 6 0 -1.214857 -0.000000 1.669565<br>8 6 0 0.982840 0.714075 1.669565<br>9 6 0 0.982840 -0.714075 1.669565<br>10 6 0 -0.375412 -1.155398 1.669565<br>11 6 0 -0.375412 1.155398 1.669565<br>12 1 0 -2.297208 -0.000000 -1.659531<br>13 1 0 1.858480 1.350265 -1.659531<br>14 1 0 1.858480 -1.350265 -1.659531<br>15 1 0 -0.709876 -2.184775 -1.659531<br>16 1 0 -0.709876 2.184775 -1.659531<br>17 1 0 -2.297208 -0.000000 1.659531<br>18 1 0 1.858480 1.350265 1.659531<br>19 1 0 1.858480 -1.350265 1.659531<br>20 1 0 -0.709876 -2.184775 1.659531<br>21 1 0 -0.709876 2.184775 1.659531<br><br>Rotational constants (GHZ):<br>2.0528214 1.0329299 1.0151710
D5h-Fc-3b-1	3	1 26 0 -0.000000 -0.000000 -0.000000<br>2 6 0 -1.214857 -0.000000 -1.669565<br>3 6 0 0.982840 0.714075 -1.669565<br>4 6 0 0.982840 -0.714075 -1.669565<br>5 6 0 -0.375412 -1.155398 -1.669565<br>6 6 0 -0.375412 1.155398 -1.669565<br>7 6 0 -1.214857 -0.000000 1.669565<br>8 6 0 0.982840 0.714075 1.669565<br>9 6 0 0.982840 -0.714075 1.669565<br>10 6 0 -0.375412 -1.155398 1.669565<br>11 6 0 -0.375412 1.155398 1.669565<br>12 1 0 -2.297208 -0.000000 -1.659531<br>13 1 0 1.858480 1.350265 -1.659531<br>14 1 0 1.858480 -1.350265 -1.659531<br>15 1 0 -0.709876 -2.184775 -1.659531<br>16 1 0 -0.709876 2.184775 -1.659531<br>17 1 0 -2.297208 -0.000000 1.659531<br>18 1 0 1.858480 1.350265 1.659531<br>19 1 0 1.858480 -1.350265 1.659531<br>20 1 0 -0.709876 -2.184775 1.659531<br>21 1 0 -0.709876 2.184775 1.659531<br><br>Rotational constants (GHZ):<br>2.0544999 1.0310845 1.0167426
D5h-Fc-3b-2	3	1 26 0 -0.000000 -0.000000 -0.000000<br>2 6 0 -1.214857 -0.000000 -1.669565<br>3 6 0 0.982840 0.714075 -1.669565

		4 6 0 0.982840 -0.714075 -1.669565<br>5 6 0 -0.375412 -1.155398 -1.669565<br>6 6 0 -0.375412 1.155398 -1.669565<br>7 6 0 -1.214857 -0.000000 1.669565<br>8 6 0 0.982840 0.714075 1.669565<br>9 6 0 0.982840 -0.714075 1.669565<br>10 6 0 -0.375412 -1.155398 1.669565<br>11 6 0 -0.375412 1.155398 1.669565<br>12 1 0 -2.297208 -0.000000 -1.659531<br>13 1 0 1.858480 1.350265 -1.659531<br>14 1 0 1.858480 -1.350265 -1.659531<br>15 1 0 -0.709876 -2.184775 -1.659531<br>16 1 0 -0.709876 2.184775 -1.659531<br>17 1 0 -2.297208 -0.000000 1.659531<br>18 1 0 1.858480 1.350265 1.659531<br>19 1 0 1.858480 -1.350265 1.659531<br>20 1 0 -0.709876 -2.184775 1.659531<br>21 1 0 -0.709876 2.184775 1.659531<br><br>Rotational constants (GHZ):<br>2.0554529 1.0268661 1.0202214
D5h-Fc-3b-3	3	1 26 0 -0.000000 -0.000000 -0.000000<br>2 6 0 -1.214857 -0.000000 -1.669565<br>3 6 0 0.982840 0.714075 -1.669565<br>4 6 0 0.982840 -0.714075 -1.669565<br>5 6 0 -0.375412 -1.155398 -1.669565<br>6 6 0 -0.375412 1.155398 -1.669565<br>7 6 0 -1.214857 -0.000000 1.669565<br>8 6 0 0.982840 0.714075 1.669565<br>9 6 0 0.982840 -0.714075 1.669565<br>10 6 0 -0.375412 -1.155398 1.669565<br>11 6 0 -0.375412 1.155398 1.669565<br>12 1 0 -2.297208 -0.000000 -1.659531<br>13 1 0 1.858480 1.350265 -1.659531<br>14 1 0 1.858480 -1.350265 -1.659531<br>15 1 0 -0.709876 -2.184775 -1.659531<br>16 1 0 -0.709876 2.184775 -1.659531<br>17 1 0 -2.297208 -0.000000 1.659531<br>18 1 0 1.858480 1.350265 1.659531<br>19 1 0 1.858480 -1.350265 1.659531<br>20 1 0 -0.709876 -2.184775 1.659531<br>21 1 0 -0.709876 2.184775 1.659531<br><br>Rotational constants (GHZ):<br>2.0560844 1.0318571 1.0149304
D5h-Fc-04a	4	1 26 0 -0.000000 -0.000000 -0.000000<br>2 6 0 -1.214857 -0.000000 -1.669565<br>3 6 0 0.982840 0.714075 -1.669565<br>4 6 0 0.982840 -0.714075 -1.669565<br>5 6 0 -0.375412 -1.155398 -1.669565<br>6 6 0 -0.375412 1.155398 -1.669565<br>7 6 0 -1.214857 -0.000000 1.669565<br>8 6 0 0.982840 0.714075 1.669565<br>9 6 0 0.982840 -0.714075 1.669565<br>10 6 0 -0.375412 -1.155398 1.669565<br>11 6 0 -0.375412 1.155398 1.669565

		12 1 0 -2.297208 -0.000000 -1.659531<br>13 1 0 1.858480 1.350265 -1.659531<br>14 1 0 1.858480 -1.350265 -1.659531<br>15 1 0 -0.709876 -2.184775 -1.659531<br>16 1 0 -0.709876 2.184775 -1.659531<br>17 1 0 -2.297208 -0.000000 1.659531<br>18 1 0 1.858480 1.350265 1.659531<br>19 1 0 1.858480 -1.350265 1.659531<br>20 1 0 -0.709876 -2.184775 1.659531<br>21 1 0 -0.709876 2.184775 1.659531<br>Rotational constants (GHZ):<br>2.0098869 1.0185348 1.0075798
D5h-Fc-05a	5	1 26 0 0.000000 0.000000 0.000000<br>2 6 0 0.000000 1.214857 1.669565<br>3 6 0 -0.714075 -0.982840 1.669565<br>4 6 0 0.714075 -0.982840 1.669565<br>5 6 0 1.155398 0.375411 1.669565<br>6 6 0 -1.155398 0.375411 1.669565<br>7 6 0 0.000000 1.214857 -1.669565<br>8 6 0 -0.714075 -0.982840 -1.669565<br>9 6 0 0.714075 -0.982840 -1.669565<br>10 6 0 1.155398 0.375411 -1.669565<br>11 6 0 -1.155398 0.375411 -1.669565<br>12 1 0 0.000000 2.297208 1.659531<br>13 1 0 -1.350265 -1.858480 1.659531<br>14 1 0 1.350265 -1.858480 1.659531<br>15 1 0 2.184775 0.709876 1.659531<br>16 1 0 -2.184775 0.709876 1.659531<br>17 1 0 0.000000 2.297208 -1.659531<br>18 1 0 -1.350265 -1.858480 -1.659531<br>19 1 0 1.350265 -1.858480 -1.659531<br>20 1 0 2.184775 0.709876 -1.659531<br>21 1 0 -2.184775 0.709876 -1.659531<br><br>Rotational constants (GHZ):<br>1.9676705 1.0024140 1.0024140
D5h-Fc-06a	6	1 26 -0.000000000 0.000000000 0.000000000<br>2 6 -0.000513478 0.000000000 -0.000115203<br>3 6 0.000415412 0.000301815 -0.000115203<br>4 6 0.000415412 -0.000301815 -0.000115203<br>5 6 -0.000158673 -0.000488347 -0.000115203<br>6 6 -0.000158673 0.000488347 -0.000115203<br>7 6 -0.000513478 -0.000000000 0.000115203<br>8 6 0.000415412 0.000301815 0.000115203<br>9 6 0.000415412 -0.000301815 0.000115203<br>10 6 -0.000158673 -0.000488347 0.000115203<br>11 6 -0.000158673 0.000488347 0.000115203<br>12 1 0.000033876 -0.000000000 -0.000000433<br>13 1 -0.000027407 -0.000019912 -0.000000433<br>14 1 -0.000027407 0.000019912 -0.000000433<br>15 1 0.000010468 0.000032218 -0.000000433<br>16 1 0.000010468 -0.000032218 -0.000000433<br>17 1 0.000033876 -0.000000000 0.000000433<br>18 1 -0.000027407 -0.000019912 0.000000433<br>19 1 -0.000027407 0.000019912 0.000000433<br>20 1 0.000010468 0.000032218 0.000000433

		21 1 0.000010468 -0.000032218 0.000000433<br><br>Rotational constants (GHZ):<br>1.9284775 0.9965502 0.9863972
D5h-Fc-6b-1	6	1 26 0 0.000000 0.000000 0.000000<br>2 6 0 0.000000 1.214857 1.669565<br>3 6 0 -0.714075 -0.982840 1.669565<br>4 6 0 0.714075 -0.982840 1.669565<br>5 6 0 1.155398 0.375411 1.669565<br>6 6 0 -1.155398 0.375411 1.669565<br>7 6 0 0.000000 1.214857 -1.669565<br>8 6 0 -0.714075 -0.982840 -1.669565<br>9 6 0 0.714075 -0.982840 -1.669565<br>10 6 0 1.155398 0.375411 -1.669565<br>11 6 0 -1.155398 0.375411 -1.669565<br>12 1 0 0.000000 2.297208 1.659531<br>13 1 0 -1.350265 -1.858480 1.659531<br>14 1 0 1.350265 -1.858480 1.659531<br>15 1 0 2.184775 0.709876 1.659531<br>16 1 0 -2.184775 0.709876 1.659531<br>17 1 0 0.000000 2.297208 -1.659531<br>18 1 0 -1.350265 -1.858480 -1.659531<br>19 1 0 1.350265 -1.858480 -1.659531<br>20 1 0 2.184775 0.709876 -1.659531<br>21 1 0 -2.184775 0.709876 -1.659531<br><br>Rotational constants (GHZ):<br>1.9307519 0.9960311 0.9856414
D5h-Fc-6b-2	6	1 26 0 0.000000 0.000000 0.000000<br>2 6 0 0.000000 1.214857 1.669565<br>3 6 0 -0.714075 -0.982840 1.669565<br>4 6 0 0.714075 -0.982840 1.669565<br>5 6 0 1.155398 0.375411 1.669565<br>6 6 0 -1.155398 0.375411 1.669565<br>7 6 0 0.000000 1.214857 -1.669565<br>8 6 0 -0.714075 -0.982840 -1.669565<br>9 6 0 0.714075 -0.982840 -1.669565<br>10 6 0 1.155398 0.375411 -1.669565<br>11 6 0 -1.155398 0.375411 -1.669565<br>12 1 0 0.000000 2.297208 1.659531<br>13 1 0 -1.350265 -1.858480 1.659531<br>14 1 0 1.350265 -1.858480 1.659531<br>15 1 0 2.184775 0.709876 1.659531<br>16 1 0 -2.184775 0.709876 1.659531<br>17 1 0 0.000000 2.297208 -1.659531<br>18 1 0 -1.350265 -1.858480 -1.659531<br>19 1 0 1.350265 -1.858480 -1.659531<br>20 1 0 2.184775 0.709876 -1.659531<br>21 1 0 -2.184775 0.709876 -1.659531<br><br>Rotational constants (GHZ):<br>1.9299303 0.9990032 0.9833462
D5h-Fc-6b-3	6	1 26 0 0.000000 0.000000 0.000000<br>2 6 0 0.000000 1.214857 1.669565<br>3 6 0 -0.714075 -0.982840 1.669565<br>4 6 0 0.714075 -0.982840 1.669565

		5 6 0 1.155398 0.375411 1.669565<br>6 6 0 -1.155398 0.375411 1.669565<br>7 6 0 0.000000 1.214857 -1.669565<br>8 6 0 -0.714075 -0.982840 -1.669565<br>9 6 0 0.714075 -0.982840 -1.669565<br>10 6 0 1.155398 0.375411 -1.669565<br>11 6 0 -1.155398 0.375411 -1.669565<br>12 1 0 0.000000 2.297208 1.659531<br>13 1 0 -1.350265 -1.858480 1.659531<br>14 1 0 1.350265 -1.858480 1.659531<br>15 1 0 2.184775 0.709876 1.659531<br>16 1 0 -2.184775 0.709876 1.659531<br>17 1 0 0.000000 2.297208 -1.659531<br>18 1 0 -1.350265 -1.858480 -1.659531<br>19 1 0 1.350265 -1.858480 -1.659531<br>20 1 0 2.184775 0.709876 -1.659531<br>21 1 0 -2.184775 0.709876 -1.659531<br><br>Rotational constants (GHZ):<br>1.9293971 0.9934903 0.9891276
D5h-Fc-6b-4	6	1 26 0 0.000000 0.000000 0.000000<br>2 6 0 0.000000 1.214857 1.669565<br>3 6 0 -0.714075 -0.982840 1.669565<br>4 6 0 0.714075 -0.982840 1.669565<br>5 6 0 1.155398 0.375411 1.669565<br>6 6 0 -1.155398 0.375411 1.669565<br>7 6 0 0.000000 1.214857 -1.669565<br>8 6 0 -0.714075 -0.982840 -1.669565<br>9 6 0 0.714075 -0.982840 -1.669565<br>10 6 0 1.155398 0.375411 -1.669565<br>11 6 0 -1.155398 0.375411 -1.669565<br>12 1 0 0.000000 2.297208 1.659531<br>13 1 0 -1.350265 -1.858480 1.659531<br>14 1 0 1.350265 -1.858480 1.659531<br>15 1 0 2.184775 0.709876 1.659531<br>16 1 0 -2.184775 0.709876 1.659531<br>17 1 0 0.000000 2.297208 -1.659531<br>18 1 0 -1.350265 -1.858480 -1.659531<br>19 1 0 1.350265 -1.858480 -1.659531<br>20 1 0 2.184775 0.709876 -1.659531<br>21 1 0 -2.184775 0.709876 -1.659531<br><br>Rotational constants (GHZ):<br>1.9289490 1.0048496 0.9775280
D5h-Fc-6b-5	6	1 26 0 0.000000 0.000000 0.000000<br>2 6 0 0.000000 1.214857 1.669565<br>3 6 0 -0.714075 -0.982840 1.669565<br>4 6 0 0.714075 -0.982840 1.669565<br>5 6 0 1.155398 0.375411 1.669565<br>6 6 0 -1.155398 0.375411 1.669565<br>7 6 0 0.000000 1.214857 -1.669565<br>8 6 0 -0.714075 -0.982840 -1.669565<br>9 6 0 0.714075 -0.982840 -1.669565<br>10 6 0 1.155398 0.375411 -1.669565<br>11 6 0 -1.155398 0.375411 -1.669565<br>12 1 0 0.000000 2.297208 1.659531

		13 1 0 -1.350265 -1.858480 1.659531<br>14 1 0 1.350265 -1.858480 1.659531<br>15 1 0 2.184775 0.709876 1.659531<br>16 1 0 -2.184775 0.709876 1.659531<br>17 1 0 0.000000 2.297208 -1.659531<br>18 1 0 -1.350265 -1.858480 -1.659531<br>19 1 0 1.350265 -1.858480 -1.659531<br>20 1 0 2.184775 0.709876 -1.659531<br>21 1 0 -2.184775 0.709876 -1.659531<br><br>Rotational constants (GHZ):<br>1.9287773 0.9962880 0.9859051
D5h-Fc-6b-6	6	1 26 0 0.000000 0.000000 0.000000<br>2 6 0 0.000000 1.214857 1.669565<br>3 6 0 -0.714075 -0.982840 1.669565<br>4 6 0 0.714075 -0.982840 1.669565<br>5 6 0 1.155398 0.375411 1.669565<br>6 6 0 -1.155398 0.375411 1.669565<br>7 6 0 0.000000 1.214857 -1.669565<br>8 6 0 -0.714075 -0.982840 -1.669565<br>9 6 0 0.714075 -0.982840 -1.669565<br>10 6 0 1.155398 0.375411 -1.669565<br>11 6 0 -1.155398 0.375411 -1.669565<br>12 1 0 0.000000 2.297208 1.659531<br>13 1 0 -1.350265 -1.858480 1.659531<br>14 1 0 1.350265 -1.858480 1.659531<br>15 1 0 2.184775 0.709876 1.659531<br>16 1 0 -2.184775 0.709876 1.659531<br>17 1 0 0.000000 2.297208 -1.659531<br>18 1 0 -1.350265 -1.858480 -1.659531<br>19 1 0 1.350265 -1.858480 -1.659531<br>20 1 0 2.184775 0.709876 -1.659531<br>21 1 0 -2.184775 0.709876 -1.659531<br><br>Rotational constants (GHZ):<br>1.9284631 1.0024542 0.9801675
D5h-Fc-7b-1,2	7	1 26 0 0.000000 0.000000 0.000000<br>2 6 0 0.000000 1.214857 1.669565<br>3 6 0 -0.714075 -0.982840 1.669565<br>4 6 0 0.714075 -0.982840 1.669565<br>5 6 0 1.155398 0.375411 1.669565<br>6 6 0 -1.155398 0.375411 1.669565<br>7 6 0 0.000000 1.214857 -1.669565<br>8 6 0 -0.714075 -0.982840 -1.669565<br>9 6 0 0.714075 -0.982840 -1.669565<br>10 6 0 1.155398 0.375411 -1.669565<br>11 6 0 -1.155398 0.375411 -1.669565<br>12 1 0 0.000000 2.297208 1.659531<br>13 1 0 -1.350265 -1.858480 1.659531<br>14 1 0 1.350265 -1.858480 1.659531<br>15 1 0 2.184775 0.709876 1.659531<br>16 1 0 -2.184775 0.709876 1.659531<br>17 1 0 0.000000 2.297208 -1.659531<br>18 1 0 -1.350265 -1.858480 -1.659531<br>19 1 0 1.350265 -1.858480 -1.659531<br>20 1 0 2.184775 0.709876 -1.659531

		21 1 0 -2.184775 0.709876 -1.659531<br>Rotational constants (GHZ):<br>1.8911897 0.9836675 0.9778658
D5h-Fc-7b-1,3	7	1 26 0 0.000000 0.000000 0.000000<br>2 6 0 0.000000 1.214857 1.669565<br>3 6 0 -0.714075 -0.982840 1.669565<br>4 6 0 0.714075 -0.982840 1.669565<br>5 6 0 1.155398 0.375411 1.669565<br>6 6 0 -1.155398 0.375411 1.669565<br>7 6 0 0.000000 1.214857 -1.669565<br>8 6 0 -0.714075 -0.982840 -1.669565<br>9 6 0 0.714075 -0.982840 -1.669565<br>10 6 0 1.155398 0.375411 -1.669565<br>11 6 0 -1.155398 0.375411 -1.669565<br>12 1 0 0.000000 2.297208 1.659531<br>13 1 0 -1.350265 -1.858480 1.659531<br>14 1 0 1.350265 -1.858480 1.659531<br>15 1 0 2.184775 0.709876 1.659531<br>16 1 0 -2.184775 0.709876 1.659531<br>17 1 0 0.000000 2.297208 -1.659531<br>18 1 0 -1.350265 -1.858480 -1.659531<br>19 1 0 1.350265 -1.858480 -1.659531<br>20 1 0 2.184775 0.709876 -1.659531<br>21 1 0 -2.184775 0.709876 -1.659531<br><br>Rotational constants (GHZ):<br>1.8897739 0.9891100 0.9726890
D5h-Fc-8c-1,2	8	1 26 0 0.000000 0.000000 0.000000<br>2 6 0 0.000000 1.214857 1.669565<br>3 6 0 -0.714075 -0.982840 1.669565<br>4 6 0 0.714075 -0.982840 1.669565<br>5 6 0 1.155398 0.375411 1.669565<br>6 6 0 -1.155398 0.375411 1.669565<br>7 6 0 0.000000 1.214857 -1.669565<br>8 6 0 -0.714075 -0.982840 -1.669565<br>9 6 0 0.714075 -0.982840 -1.669565<br>10 6 0 1.155398 0.375411 -1.669565<br>11 6 0 -1.155398 0.375411 -1.669565<br>12 1 0 0.000000 2.297208 1.659531<br>13 1 0 -1.350265 -1.858480 1.659531<br>14 1 0 1.350265 -1.858480 1.659531<br>15 1 0 2.184775 0.709876 1.659531<br>16 1 0 -2.184775 0.709876 1.659531<br>17 1 0 0.000000 2.297208 -1.659531<br>18 1 0 -1.350265 -1.858480 -1.659531<br>19 1 0 1.350265 -1.858480 -1.659531<br>20 1 0 2.184775 0.709876 -1.659531<br>21 1 0 -2.184775 0.709876 -1.659531<br><br>Rotational constants (GHZ):<br>1.8543117 0.9736882 0.9671451
D5h-Fc-8c-1,3	8	1 26 0 0.000000 0.000000 0.000000<br>2 6 0 0.000000 1.214857 1.669565<br>3 6 0 -0.714075 -0.982840 1.669565<br>4 6 0 0.714075 -0.982840 1.669565<br>5 6 0 1.155398 0.375411 1.669565

		6 6 0 -1.155398 0.375411 1.669565<br>7 6 0 0.000000 1.214857 -1.669565<br>8 6 0 -0.714075 -0.982840 -1.669565<br>9 6 0 0.714075 -0.982840 -1.669565<br>10 6 0 1.155398 0.375411 -1.669565<br>11 6 0 -1.155398 0.375411 -1.669565<br>12 1 0 0.000000 2.297208 1.659531<br>13 1 0 -1.350265 -1.858480 1.659531<br>14 1 0 1.350265 -1.858480 1.659531<br>15 1 0 2.184775 0.709876 1.659531<br>16 1 0 -2.184775 0.709876 1.659531<br>17 1 0 0.000000 2.297208 -1.659531<br>18 1 0 -1.350265 -1.858480 -1.659531<br>19 1 0 1.350265 -1.858480 -1.659531<br>20 1 0 2.184775 0.709876 -1.659531<br>21 1 0 -2.184775 0.709876 -1.659531<br><br>Rotational constants (GHZ):<br>1.8529829 0.9785135 0.9625635
D5h-Fc-9	9	1 26 0 0.000000 0.000000 0.000000<br>2 6 0 0.000000 1.214857 1.669565<br>3 6 0 -0.714075 -0.982840 1.669565<br>4 6 0 0.714075 -0.982840 1.669565<br>5 6 0 1.155398 0.375411 1.669565<br>6 6 0 -1.155398 0.375411 1.669565<br>7 6 0 0.000000 1.214857 -1.669565<br>8 6 0 -0.714075 -0.982840 -1.669565<br>9 6 0 0.714075 -0.982840 -1.669565<br>10 6 0 1.155398 0.375411 -1.669565<br>11 6 0 -1.155398 0.375411 -1.669565<br>12 1 0 0.000000 2.297208 1.659531<br>13 1 0 -1.350265 -1.858480 1.659531<br>14 1 0 1.350265 -1.858480 1.659531<br>15 1 0 2.184775 0.709876 1.659531<br>16 1 0 -2.184775 0.709876 1.659531<br>17 1 0 0.000000 2.297208 -1.659531<br>18 1 0 -1.350265 -1.858480 -1.659531<br>19 1 0 1.350265 -1.858480 -1.659531<br>20 1 0 2.184775 0.709876 -1.659531<br>21 1 0 -2.184775 0.709876 -1.659531<br><br>Rotational constants (GHZ):<br>1.8179377 0.9653186 0.9554690
D5h-Fc-10	10	1 26 0 0.000000 0.000000 0.000000<br>2 6 0 0.000000 1.214857 1.669565<br>3 6 0 -0.714075 -0.982840 1.669565<br>4 6 0 0.714075 -0.982840 1.669565<br>5 6 0 1.155398 0.375411 1.669565<br>6 6 0 -1.155398 0.375411 1.669565<br>7 6 0 0.000000 1.214857 -1.669565<br>8 6 0 -0.714075 -0.982840 -1.669565<br>9 6 0 0.714075 -0.982840 -1.669565<br>10 6 0 1.155398 0.375411 -1.669565<br>11 6 0 -1.155398 0.375411 -1.669565<br>12 1 0 0.000000 2.297208 1.659531<br>13 1 0 -1.350265 -1.858480 1.659531

		14 1 0 1.350265 -1.858480 1.659531<br>15 1 0 2.184775 0.709876 1.659531<br>16 1 0 -2.184775 0.709876 1.659531<br>17 1 0 0.000000 2.297208 -1.659531<br>18 1 0 -1.350265 -1.858480 -1.659531<br>19 1 0 1.350265 -1.858480 -1.659531<br>20 1 0 2.184775 0.709876 -1.659531<br>21 1 0 -2.184775 0.709876 -1.659531<br><br>Rotational constants (GHZ):<br>1.7833168 0.9505694 0.9505694
D5h-Fc	10	1 26 0 0.000000 -0.000000 0.000000<br>2 6 0 1.669565 -0.000000 -1.214857<br>3 6 0 1.669565 0.714075 0.982840<br>4 6 0 1.669565 -0.714075 0.982840<br>5 6 0 1.669565 -1.155398 -0.375412<br>6 6 0 1.669565 1.155398 -0.375412<br>7 6 0 -1.669565 0.000000 -1.214857<br>8 6 0 -1.669565 0.714075 0.982840<br>9 6 0 -1.669565 -0.714075 0.982840<br>10 6 0 -1.669565 -1.155398 -0.375412<br>11 6 0 -1.669565 1.155398 -0.375412<br>12 1 0 1.659531 -0.000000 -2.297208<br>13 1 0 1.659531 1.350265 1.858480<br>14 1 0 1.659531 -1.350265 1.858480<br>15 1 0 1.659531 -2.184775 -0.709876<br>16 1 0 1.659531 2.184775 -0.709876<br>17 1 0 -1.659531 0.000000 -2.297208<br>18 1 0 -1.659531 1.350265 1.858480<br>19 1 0 -1.659531 -1.350265 1.858480<br>20 1 0 -1.659531 -2.184775 -0.709876<br>21 1 0 -1.659531 2.184775 -0.709876<br><br>Rotational constants (GHZ):<br>1.7833162 0.9505693 0.9505692

Table S2. DFT-calculated far-IR modes (400–600 cm^{-1}) for selected nD-Fc (cm^{-1})*.

nd-Fc	Mode	ν_{DFT} (cm^{-1})	IR(Int)	ν_{AI} (cm^{-1})	$\Delta\nu$ (cm^{-1})
D5h_Fc-0	1	471.0703	17.6677	457.4356	-11.63465238
D5h_Fc-0	2	488.4522	22.317	483.5529	-4.899314286
D5h_Fc-0	3	488.4522	22.3176	484.0114	-4.440804762
D5h_Fc-1	1	465.8918	18.3258	457.4356	-8.456152381
D5h_Fc-1	2	487.1264	22.4909	483.5529	-3.573514286
D5h_Fc-1	3	487.3981	22.2382	484.0114	-3.386704762
D5h_Fc-2a-1,2	1	463.6818	18.8932	457.4356	-6.246152381

D5h_Fc-2a-1,2	2	485.9301	22.4537	483.5529	-2.377214286
D5h_Fc-2a-1,2	3	486.3801	22.3415	484.0114	-2.368704762
D5h_Fc-2a-1,3	1	462.1501	18.9496	457.4356	-4.714452381
D5h_Fc-2a-1,3	2	485.7763	22.6072	483.5529	-2.223414286
D5h_Fc-2a-1,3	3	486.3141	22.3321	484.0114	-2.302704762
D5h_Fc-2b-1	1	463.1424	18.763	457.4356	-5.706752381
D5h_Fc-2b-1	2	485.7929	22.6656	483.5529	-2.240014286
D5h_Fc-2b-1	3	486.0376	22.2977	484.0114	-2.026204762
D5h_Fc-2b-2	1	462.8995	18.9015	457.4356	-5.463852381
D5h_Fc-2b-2	2	485.9126	22.4118	483.5529	-2.359714286
D5h_Fc-2b-2	3	486.0268	22.465	484.0114	-2.015404762
D5h_Fc-2b-3	1	462.3885	19.1192	457.4356	-4.952852381
D5h_Fc-2b-3	2	485.8071	22.6278	483.5529	-2.254214286
D5h_Fc-2b-3	3	486.4348	22.152	484.0114	-2.423404762
D5h_Fc-3b-1	1	460.9924	19.2949	457.4356	-3.556752381
D5h_Fc-3b-1	2	484.6287	22.5696	483.5529	-1.075814286
D5h_Fc-3b-1	3	484.8661	22.4855	484.0114	-0.854704762
D5h_Fc-3b-2	1	460.354	19.6002	457.4356	-2.918352381
D5h_Fc-3b-2	2	484.6222	22.5309	483.5529	-1.069314286
D5h_Fc-3b-2	3	485.1941	22.3685	484.0114	-1.182704762
D5h_Fc-3b-3	1	459.9197	19.7909	457.4356	-2.484052381
D5h_Fc-3b-3	2	484.5484	22.6336	483.5529	-0.995514286
D5h_Fc-3b-3	3	485.5034	22.1861	484.0114	-1.492004762
D5h_Fc-03a	1	459.4638	19.5337	457.4356	-2.028152381
D5h_Fc-03a	2	484.5131	22.6411	483.5529	-0.960214286
D5h_Fc-03a	3	485.3642	22.4342	484.0114	-1.352804762
D5h_Fc-04a	1	457.6633	20.0527	457.4356	-0.227652381
D5h_Fc-04a	2	483.7248	22.6707	483.5529	-0.171914286
D5h_Fc-04a	3	484.3372	22.5666	484.0114	-0.325804762
D5h_Fc-05a	1	455.466	20.6169	457.4356	1.969647619
D5h_Fc-05a	2	483.1495	22.721	483.5529	0.403385714

D5h_Fc-05a	3	483.1495	22.7204	484.0114	0.861895238
D5h_Fc-06a	1	452.3004	21.2148	457.4356	5.135247619
D5h_Fc-06a	2	481.6899	22.9113	483.5529	1.862985714
D5h_Fc-06a	3	481.8028	22.7262	484.0114	2.208595238
D5h_Fc-06b-1	1	451.9443	21.3555	457.4356	5.491347619
D5h_Fc-06b-1	2	481.1398	22.7878	483.5529	2.413085714
D5h_Fc-06b-1	3	481.7527	22.7008	484.0114	2.258695238
D5h_Fc-06b-2	1	452.3474	21.1347	457.4356	5.088247619
D5h_Fc-06b-2	2	480.8942	22.8871	483.5529	2.658685714
D5h_Fc-06b-2	3	481.8659	22.7045	484.0114	2.145495238
D5h_Fc-06b-3	1	452.6345	21.001	457.4356	4.801147619
D5h_Fc-06b-3	2	481.1523	22.8693	483.5529	2.400585714
D5h_Fc-06b-3	3	481.4861	22.7761	484.0114	2.525295238
D5h_Fc-06b-4	1	450.612	21.3349	457.4356	6.823647619
D5h_Fc-06b-4	2	480.8882	22.9674	483.5529	2.664685714
D5h_Fc-06b-4	3	481.8077	22.6882	484.0114	2.203695238
D5h_Fc-06b-5	1	450.825	21.2914	457.4356	6.610647619
D5h_Fc-06b-5	2	481.1199	22.8187	483.5529	2.432985714
D5h_Fc-06b-5	3	481.4356	22.8521	484.0114	2.575795238
D5h_Fc-06b-6	1	450.9685	21.1781	457.4356	6.467147619
D5h_Fc-06b-6	2	480.8253	22.9958	483.5529	2.727585714
D5h_Fc-06b-6	3	481.7134	22.7221	484.0114	2.297995238

Table S3. DFT calculated IR three signature vibrational bands in the–400 cm^{-1} region of deuterated Fc derivatives (cm^{-1}).*

nD	Derivative	$\tilde{\nu}_1(a_2'')$ /cm^{-1}	$\tilde{\nu}_2(e_1')$ /cm^{-1}	$\tilde{\nu}_3(e_1')$ /cm^{-1}	$\Delta(e_1')$ /cm^{-1}
**0**	**D5h-Fc-0**	469.07	*488.45*	*488.45*	*0.00*
**1**	**D5h-Fc-1**	465.89	487.13	487.40	**0.27**
**2**	**D5h-Fc-2a-1,2**	463.68	485.93	486.38	**0.45**
**2**	**D5h-Fc-2a-1,3**	462.15	485.78	486.31	**0.54**
**2**	**D5h-Fc-2b-1**	463.14	485.79	486.04	**0.24**
**2**	**D5h-Fc-2b-2**	462.90	485.91	486.03	**0.11**

**2**	**D5h-Fc-2b-3**	462.39	485.81	486.43	**0.63**
**3**	**D5h-Fc-03a**	459.46	484.51	485.36	**0.85**
**3**	**D5h-Fc-3b-1**	460.99	484.63	484.87	**0.24**
**3**	**D5h-Fc-3b-2**	460.35	484.62	485.19	**0.57**
**3**	**D5h-Fc-3b-3**	459.92	484.55	485.50	**0.95**
**4**	**D5h-Fc-04a**	457.66	483.72	484.34	**0.61**
**5**	**D5h-Fc-05a**	455.47	*483.15*	*483.15*	*0.00*
**6**	**D5h-Fc-06a**	452.30	481.69	481.80	**0.11**
**6**	**D5h-Fc-06b-1**	451.94	481.14	481.75	**0.61**
**6**	**D5h-Fc-06b-2**	452.35	480.89	481.87	**0.97**
**6**	**D5h-Fc-06b-3**	452.63	481.15	481.49	**0.33**
**6**	**D5h-Fc-06b-4**	450.61	480.89	481.81	**0.92**
**6**	**D5h-Fc-06b-5**	450.83	481.12	481.44	**0.32**
**6**	**D5h-Fc-06b-6**	450.97	480.83	481.71	**0.89**
**7**	**D5h-Fc-07b-1,2**	449.99	480.24	480.65	**0.40**
**7**	**D5h-Fc-07b-1,3**	448.60	480.16	480.52	**0.36**
**8**	**D5h-Fc-08c-1,2**	447.12	479.12	479.29	**0.18**
**8**	**D5h-Fc-08c-1,3**	445.83	478.67	479.42	**0.75**
**9**	**D5h-Fc-09**	443.88	477.69	478.26	**0.57**
**10**	**D5h-Fc-10**	441.53	*476.94*	*476.94*	*0.00*

*AI helped table format from provided results.

Table S4. The physics based AI-predicted IR three vibrational bands in the 400–600 cm^{-1} region of deuterated Fc derivatives (cm^{-1}).

nD	Derivative	$\tilde{\nu}_1(a_2'')$ /cm^{-1}	$\tilde{\nu}_2(e_1')$ /cm^{-1}	$\tilde{\nu}_3(e_1')$ /cm^{-1}	$\Delta(e_1')$ /cm^{-1}
**0**	**D5h-Fc-0**	471.20	*488.70*	*488.70*	*0.00*
**1**	**D5h-Fc-1**	466.00	483.40	488.00	**4.60**
**2**	**D5h-Fc-2a-1,2**	461.90	481.40	484.40	**3.00**
**2**	**D5h-Fc-2a-1,3**	461.80	478.50	486.40	**7.90**
**2**	**D5h-Fc-2b-1**	461.80	477.60	487.70	**10.10**
**2**	**D5h-Fc-2b-2**	461.90	481.30	484.40	**3.10**
**2**	**D5h-Fc-2b-3**	461.90	478.80	486.40	**7.60**
**3**	**D5h-Fc-b-03a**	457.70	476.40	483.50	**7.10**
**3**	**D5h-Fc-3b-1**	457.50	477.10	483.00	**5.90**
**3**	**D5h-Fc-3b-2**	457.40	478.80	481.50	**2.70**
**3**	**D5h-Fc-3b-3**	457.40	477.40	483.40	**6.00**
**4**	**D5h-Fc-04a**	453.30	474.60	479.00	**4.40**
**5**	**D5h-Fc-05a**	448.60	*472.30*	*472.30*	*0.00*
**6**	**D5h-Fc-06a**	444.40	468.50	473.50	**5.00**

**6**	**D5h-Fc-6b-1**	444.20	468.80	473.40	**4.60**
**6**	**D5h-Fc-6b-2**	444.30	467.30	474.20	**6.90**
**6**	**D5h-Fc-6b-3**	444.40	469.90	472.10	**2.20**
**6**	**D5h-Fc-6b-4**	444.50	465.70	475.40	**9.70**
**6**	**D5h-Fc-6b-5**	444.50	468.70	473.40	**4.70**
**6**	**D5h-Fc-6b-6**	444.40	466.90	474.90	**8.00**
**7**	**D5h-Fc-7b-1,2**	440.30	465.90	468.70	**2.80**
**7**	**D5h-Fc-7b-1,3**	440.40	463.30	467.20	**3.90**
**8**	**D5h-Fc-8c-1,2**	436.20	462.80	465.50	**2.70**
**8**	**D5h-Fc-8c-1,3**	436.20	460.60	464.70	**4.10**
**9**	**D5h-Fc-9**	432.20	458.60	463.50	**4.90**
**10**	**D5h-Fc-10**	424.90	*463.10*	*463.10*	*0.00*

*This IR bands of original no-deuterated eclipsed Fc are the reference bands [3]. AI helped format for AI predict results with physical based.

1. Pupyshev, V.I., Y.N. Panchenko, and N.F. Stepanov, *A new derivation for the Teller—Redlich isotopic product rule.* Vibrational Spectroscopy, 1994. **7**(2): p. 191–196.
2. Hase, Y., *Evaluation of the Teller-Redlich Product Rule for Non-linear X—Y—Z Molecules by a Programmable Calculator.* Computers & Chemistry, 1984. **8**(4): p. 299–302.
3. Mohammadi, N., et al., *Differentiation of ferrocene D5d and D5h conformers using IR spectroscopy.* Journal of Organometallic Chemistry, 2012. **713**: p. 51–59.
